# Supplementary material for: Clinical and Molecular Characterization of Classical-Like Ehlers-Danlos Syndrome Due to a Novel TNXB Variant
Source: Genes (Basel). 2019 Oct 25;10(11):843. doi: 10.3390/genes10110843 (PMC6895888; doi:10.3390/genes10110843)
Supplement: Supplementary file 1 [file genes-10-00843-s001.zip › Supplementary data.pdf]

Supplementary data

Supplementary Figure S1: Steady-state analysis of collagen in the medium and the cell layer

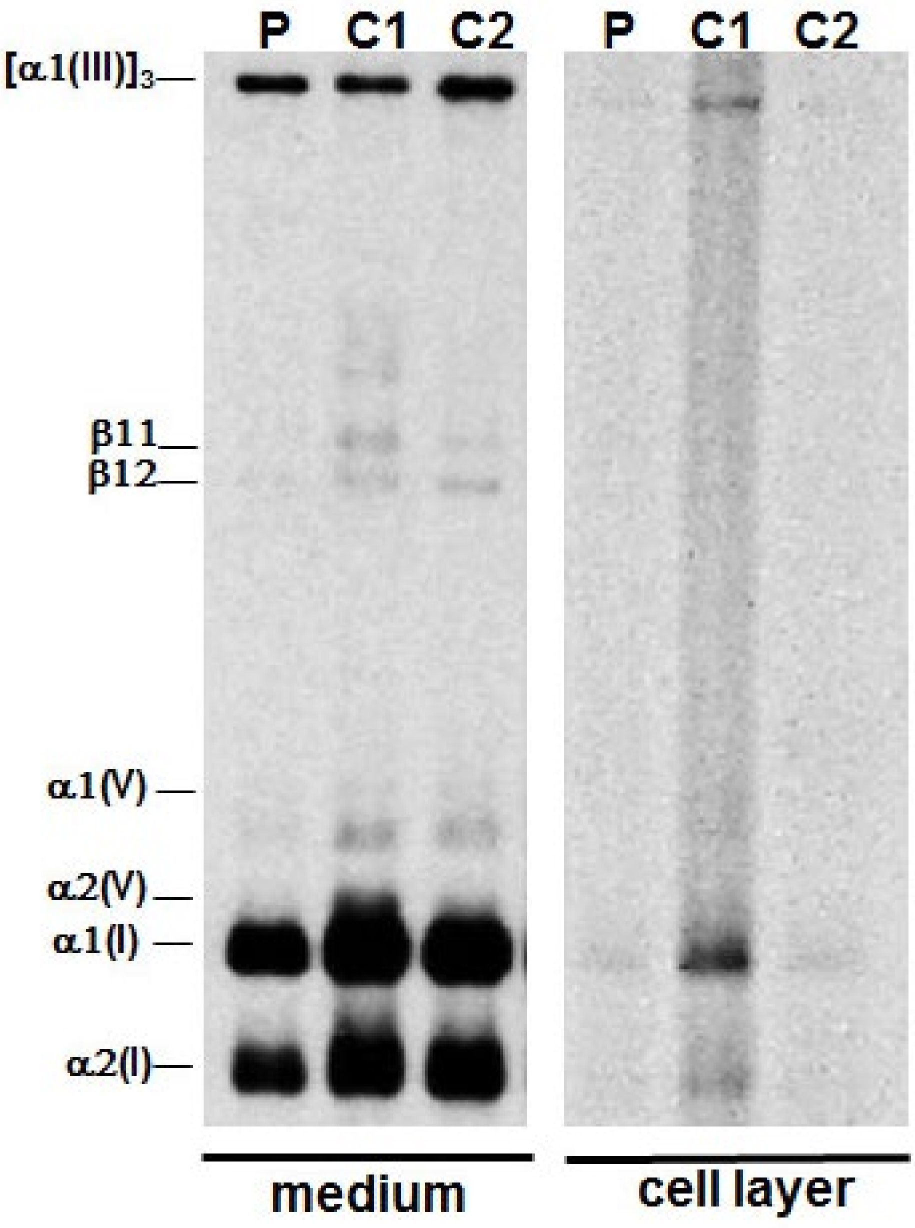

Supplementary Table S1: NGS panel for connective tissue disorders

ABCC6	NM_001171	*DSE*	NM_013352	*PPIB*	NM_000942
ACTA2	NM_001613	*DSPP*	NM_014208	*PRDM5*	NM_016599
ACVRL1	NM_000020	*EFEMP2*	NM_016938	*PRKG1*	NM_006258
ADAMTS2	NM_014244	*ELN*	NM_000501	*PYCR1*	NM_006907
ADAMTSL4	NM_019032	*ENG*	NM_000118	*RIN2*	NM_001242581
ALDH18A1	NM_002860	*FBLN5*	NM_006329	*RUNX2*	NM_001024630
ALPL	NM_000478	*FBN1*	NM_000138	*RYR1*	NM_000540
ANO5	NM_213599	*FBN2*	NM_001999	*SEC24D*	NM_014822
ATP6V0A2	NM_012463	*FGFR2*	NM_000141	*SEPN1*	NM_020451
ATP7A	NM_000052	*FGFR3*	NM_001163213	*SERPINF1*	NM_002615
B3GALT6	NM_080605	*FKBP10*	NM_0213939	*SERPINH1*	NM_001235
B4GALT7	NM_007255	*FKBP14*	NM_017946	*SKI*	NM_003036
BMP1	NM_006129	*FLCN*	NM_144997	*SLC25A2*	NM_000112
CASR	NM_001178065	*FLNA*	NM_001456	*SLC2A10*	NM_030777
CBS	NM_000071	*GLA*	NM_000169	*SLC39A13*	NM_001128225
CHRDL1	NM_001143981	*GORAB*	NM_152281	*SMAD3*	NM_005902
CHST14	NM_130468	*IFITM5*	NM_001025295	*SMAD4*	NM_005359
COL11A1	NM_001854	*LEPRE1*	NM_001243246	*SOX9*	NM_000346
(COL11A2)	NM_080680	*LRP5*	NM_002335	*SP7*	NM_001173467
COL12A1	NM_004370	*LTBP2*	NM_000428	*SPARC*	NM_003118
COL1A1	NM_000088	*LTBP4*	NM_0035732	*STAT1*	NM_007315
COL1A2	NM_000069	*MAT2A*	NM_005911	*STAT3*	NM_139278
COL2A1	NM_001844	*MED12*	NM_005120	*TGFB1*	NM_000660
COL3A1	NM_000090	*MFAP5*	NM_003480	*TGFB2*	NM_001135599
COL5A1	NM_000093	*MMP2*	NM_004530	*TGFB3*	NM_003239
COL5A2	NM_000393	*MYH11*	NM_002474	*TGFBR1*	NM_004612
COL6A1	NM_001846	*MYLK*	NM_053025	*TGFBR2*	NM_001024847
COL6A2	NM_001849	*NOTCH1*	NM_017617	*TMEM38B*	NM_018112
COL6A3	NM_004369	*NOTCH3*	NM_000435	*TRIP11*	NM_004239
COL9A1	NM_001851	*P4HB*	NM_000918	*WNT1*	NM_005430
COL9A2	NM_001852	*PLOD1*	NM_000302	*XYLT1*	NM_022166
CREB3L1	NM_052854	*PLOD2*	NM_182943	*XYLT2*	NM_022167
CRTAP	NM_006371	*PLOD3*	NM_001084	*ZDHHC9*	NM_018032
DCHS1	NM_003737	*PLS3*	NM_005032	*ZNF469*	NM_001127464

Supplementary Table S2

**Primers and PCR conditions for *TNXB* (NM_0019105.7) Sanger sequencing**		
**Exons 2-31 PCR: 95 °C, 3'; 95 °C, 30"; 58 °C. 20"; 72 °C, 45"; 72 °C, 7'; 10 °C, ∞; 32 cycles**		
**Exon 2**	**TNXBex2aF**<br>**TNXBex2aR**	GATTACTTGTGGGTGGTGGG<br>TACCTCTGAAGCAAGGACTGG
	**TNXBex2bF**<br>**TNXBex2bR**	AAGCCCCTCTTCTCAGCTTT<br>GGCTCAGATCAAACACACCA
**Exon 3**	**TNXBex3aF**<br>**TNXBex3aR**	GAGCAGACATGGCCTTATGA<br>CTCATGCCACAGTCGTCAC
	**TNXBex3bF**<br>**TNXBex3bR**	AATGATCAGGGTCGCTGTG<br>CGCAATCGGTTCCAGTGTA
	**TNXBex3cF**<br>**TNXBex3cR**	GGCGAGGACTGTGGTACG<br>CCTGAGTAGCCTGCGTCAC
	**TNXBex3dF**<br>**TNXBex3dR**	GAGAACGGCGTGTGTGTTT<br>ACAGGCACACTCCTTGCAC
	**TNXBex3eF**<br>**TNXBex3eR**	GCAGGCTACTCAGGGGAAG<br>TCCACTCTTCCTCAGGCTCA
**Exon 4**	**TNXBex4F**<br>**TNXBex4R**	GGGAGATTCTGTGCTCCTGA<br>GCCATCTGGACTCAACCAAT
**Exon 5**	**TNXBex5F**<br>**TNXBex5R**	GGACCTGTTTCGTAGGGTTG<br>ACTCAGGTGGGTGTCTGGTT
**Exon 6**	**TNXBex6F**<br>**TNXBex6R**	GCTCTTGGAAGGAAAGATGATG<br>ACAGGACTCCAGGCATCTGA
**Exon 7**	**TNXBex7F**<br>**TNXBex7R**	CAGGTGAGGGAACCAGAAGT<br>GCTGAACCTGCAATTCCTTT
**Exon 8**	**TNXBex8F**<br>**TNXBex8R**	GACCTGAAACCCCCGCAAAGAT<br>ATGCCCTGTTCTCCAGCCAGAG
**Exon 9**	**TNXBex9F**<br>**TNXBex9R**	CAGTCCCTCTGTACTCCCTCA<br>ACTTCTGGGAAGCCTGACAC
**Exon 10**	**TNXBex10F**<br>**TNXBex10R**	CCCAGAAGTTTAGCACTCGAA<br>TGGCAAAATGAGCTGAGAAG
**Exon 11**	**TNXBex11F**<br>**TNXBex11R**	GCTGTCTCAGCCTTCTCCAT<br>CAGCCTCAAGGTTCCACTG
**Exon 12**	**TNXBex12F**<br>**TNXBex12R**	TTTACTAACAGCCCCCTACCA<br>GTCAGGGAACAGAAAGACTGG
**Exon 13**	**TNXBex13F**<br>**TNXBex13R**	AACTATTCCCCATCTCAGTTCAC<br>TCAAATGAGACAGAGCAGGTG
**Exon 14**	**TNXBex14F**<br>**TNXBex14R**	GGAAATATTTTGGGGAAGCAC<br>CTGGGGCCAAATAATGGTAAT
**Exon 15**	**TNXBex15F**<br>**TNXBex15R**	GCTGCCATTACCATTATTTGG<br>GGATGATCGAAAGCAGACAGT
**Exon 16**	**TNXBex16F**<br>**TNXBex16R**	CTGGTCTTTCGATTGCTGACT<br>TGGAAAGAGAGGACTGAGGTG
**Exon 17**	**TNXBex17F**<br>**TNXBex17R**	TTCAGTGATGGGAAGCATGT<br>AGTGGTCAACCTCACAGGAAG
**Exon 18**	**TNXBex18F**<br>**TNXBex18R**	GGCCTCTCTGAACTGACCTC<br>CTGGATTTGCTCTCTCTGTCC
**Exon 19**	**TNXBex19F**<br>**TNXBex19R**	TTGAGGGACCATAAGGAAGC<br>CTGGGGCAATACCAAAGTCT
**Exon 20**	**TNXBex20F**<br>**TNXBex120R**	AGAGAATCCCAGAGTCCCTTG<br>AGTTCAGAGAGGCCCATTCTT
**Exon 21**	**TNXBex21F**<br>**TNXBex21R**	GGCCTCTCTGAACTGACCTC<br>GTGAAAGGGCACAGCAGTAA
**Exon 22**	**TNXBex22F**<br>**TNXBex22R**	GGATACCTCACCGGCTCTTA<br>TCTCCATGACATGTCTTTCCA
**Exon 23**	**TNXBex23aF**	CAATCCAGCAAATGAAGCAA

	**TNXBex23R**	CAGTCATCACCAAAGAGCAAG
**Exon 24**	**TNXBex24F**<br>**TNXBex24R**	AGCTGACCCTGGAACTTGTC<br>TCTTAGCAAGATCCCCAAGC
**Exon 25**	**TNXBex25F**<br>**TNXBex25R**	CTCTTGGGCACTTTGTGTTTT<br>CACCCAGGAAGATCTGTCAGT
**Exon 26**	**TNXBex26F**<br>**TNXBex26R**	CCTCAGAGCTTGTCATGTGTG<br>AAGCCTGCTGAATCCAAATC
**Exon 27**	**TNXBex27F**<br>**TNXBex27R**	CAGGGTGAGGGATAGGAAAG<br>ACCAAAGAGCAAGAGGTGG
**Exon 28**	**TNXBex28F**<br>**TNXBex28R**	CCCAAGAATGGACTTCTCTGA<br>TGAGGCAGGATCATTAGCAA
**Exon 29**	**TNXBex29F**<br>**TNXBex29R**	TCATATGTTGTGCGAGGGTTA<br>CAATAAATCAGTGGGTGCTGAG
**Exon 30**	**TNXBex30F**<br>**TNXBex30R**	AGGGGACACTTGCTTTCTTG<br>ACACAGAGGGACTCACTTTCG
**Exon 31**	**TNXBex31F**<br>**TNXBex31R**	GTTCTCCCTCATTCCTGTGG<br>CCTGCTCTGGACTCCTTGAT
**Exons 32-44**	***Long-range PCR (5440 bp) as template for nested-PCR***<br>**95 °C, 4'; 95 °C, 30''; 60 °C, 30''; 72 °C, 5'; 72 °C, 10'; 10 °C, ∞; 30 cycles**	
	**Intr30F**	GTGGCCTTGTCCAGATAGC
	**3'UTR-R**	ACAGCCCGGGCCAGAG
Nested-PCR: **95 °C, 3'; 95 °C, 30''; 58 °C. 20''; 72 °C, 45''; 72 °C, 7'; 10 °C, ∞; 32 cycles**		
**Exon 32**	**TNXBex32F**<br>**TNXBex32R**	GACTGGGCCTGGACCTATAA<br>AGCCCATCCATCCTCTCTC
**Exon 33**	**TNXBex33F**<br>**TNXBex33R**	TGCAAGTCCCTGGTTACAGA<br>AAGTCGCTCTGCAGATTCCT
**Exons 34-37**	**TNXBex33F**<br>**TNXBex37R**	CTGACACTGTATGGGCTGCG<br>CACAGACCCTACCTGTGGTG
**Exons 37-38**	**TNXBex37F**<br>**TNXBex38R**	TTGTCCTCCACACCAACTACA<br>ATCTCCTGTGGGACAGACAAG
**Exons 39-41**	**TNXBex38F**<br>**TNXBex41R**	TACCTGCTCAGCTTCCACAC<br>TCACAGCCTCTGCTTACCTG
**Exon 42**	**TNXBex42F**<br>**TNXBex42R**	GCATGGATGGACAGACAGAC<br>GCGTAGTGGCAGTTCCTGTA
**Exon 43**	**TNXBex43F**<br>**TNXBex43R**	CTCGGCTGCGGAGTACTACC<br>ACACTGTGGGGCTGAAACCT
**Exon 44**	**TNXBex44F**<br>**TNXBex44R**	GAGGGAGCTGGAGTTGATTTA<br>ACTGCAGTGTCATCCTCAAGA

Supplementary Table S3: Published *TNXB* pathogenic variants

Patient	Family	Sex	Age (years)	Cons.	Status	Allele 1	Type	Allele 2	Type	References
**P1**	I	F	43	+	Homozygous	Exon 15: c.5362del, p.(Thr1788Profs*100)	Frameshift-PTC	Exon 15: c.5362del, p.(Thr1788Profs*100)	Frameshift-PTC	This report
**P2**	II	F	69	-	Homozygous	Exon 8: c.3290_3291del, p.(Lys1097Argfs*48)	Frameshift-PTC	Exon 8: c.3290_3291del, p.(Lys1097Argfs*48)	Frameshift-PTC	Schalkwijk *et al.*, 2001
**P3**		F	59							
**P4**	III	F	60	-	Compound heterozygous	*TNXA* conversion[a]	Frameshift-PTC	*TNXA* conversion[a]	Frameshift-PTC	
**P5**		F	55							
**P6**		M	59							
**P7**	IV	M	57[†]	-	Homozygous	Exon 3: c.2117_2118insGT p.(Glu707*)	Frameshift-PTC	Exon 3: c.2117_2118insGT p.(Glu707*)	Frameshift-PTC	
**P8**	V	F	49[†]	-	Homozygous	Exon 8: c.3290_3291del, p.(Lys1097Argfs*48)	Frameshift-PTC	Exon 8: c.3290_3291del, p.(Lys1097Argfs*48)	Frameshift-PTC	
**P9**	VI	M	36	-	Compound heterozygous	*TNXB*/*TNXA* fusion[b]	Frameshift-PTC	*TNXA* conversion[b]	Frameshift-PTC	Voermans *et al.*, 2009
**P10**	VII	F	32	-	Compound heterozygous	Exon 3: c.903del, p.(Tyr301*)	Nonsense	Exon 42: c.12464-1G>A	Splicing	
**P11**		F	31							
**P12**	VIII	M	29	+	Homozygous	Exon 8: c.3290_3291del, p.(Lys1097Argfs*48)	Frameshift-PTC	Exon 8: c.3290_3291del, p.(Lys1097Argfs*48)	Frameshift-PTC	
**P13**	IX	F	27	-	Compound heterozygous	Exon 8: c.3290_3291del, p.(Lys1097Argfs*48)	Frameshift-PTC	*TNXB*/*TNXA* fusion[c]	Frameshift-PTC	Hendriks *et al.*, 2012
**P14**	X	F	12	-	Compound heterozygous	*TNXB*/*TNXA* fusion[d]	Frameshift-PTC	*TNXA* conversion[d]	Frameshift-PTC	
**P15**	XI	M	42	-	Compound heterozygous	*TNXB*/*TNXA* fusion[e]	Frameshift-PTC	Exon 41: c.12214C>T, p.(Arg4072Cys)	Missense	Pénisson-Besnier *et al.*, 2013
**P16**	XII	F	45	-	Compound heterozygous	Exon 6: c.2539C>T, p.(Arg847*)	Nonsense	Exon 9: c.3574C>T, p.(Gln1192*)	Nonsense	Sakiyama *et al.*, 2015
**P17**	XIII	M	13	-	Compound heterozygous	Exon 9 : c.3637G>A, p.(Val1213Ile)	Missense	Exon 22: c.7774G>A, p.(Gly2592Ser)	Missense	Mackenroth *et al., 2016*
**P18**	XIV	F	48	-	Compound heterozygous	Exon 43: c.12553C>T, p.(Arg4185*)	Nonsense	Exon 6: c.2590C>T, p.(Gln864*)	Nonsense	Demirdas *et al.*, 2017
**P19**	XV	F	23	-	Compound heterozygous	Exon 6: c.2590C>T, p.(Gln864*)	Nonsense	*TNXB*/*TNXA* fusion[f]	Frameshift-PTC	

**P20**	XVI	M	16	-	Compound heterozygous	Exon 5: c.2461C>T, p.(Arg821)*	Nonsense	*TNXA* conversion[g]	Frameshift-PTC
**P21**		M	14						
**P22**	XVII	F	12	-	Compound heterozygous	Exon 2: c.107_108delinsA, p.(Ala36Aspfs*68)	Frameshift-PTC	Exon 22: c.7826-1G>C	Splicing
**P23**		M	6						

Note: Exons and mutations numbering are based on transcript NM_019105.6; NP_061978.6; cons. Consanguinity; † patient died. PTC: Premature termination codon.

[a] Patients 4, 5, and 6 were compound heterozygous for the 120 bp deletion [c.11435_11524+30del, p.(Gly3812Phefs*11)] and the "*TNXA*-derived" missense variant [c.12174C>G, p.(Cys4058Trp)], probably due to gene conversion in both alleles;

[b] Patient 9 was compound heterozygous for a *TNXB*/*TNXA* fusion gene in one allele, characterized by the *TNXA*-derived variations: 120bp deletion [c.11435_11524+30del, p.(Gly3812Phefs*11)] and [c.12174C>G, p.(Cys4058Trp)], and a gene conversion in the other allele, characterized by the 120 bp deletion [c.11435_11524+30del, p.(Gly3812Phefs*11)];

[c] Patient 13 was compound heterozygous for a recurrent frameshift deletion in exon 8 in one allele, and a *TNXB*/*TNXA* fusion gene characterized by the *TNXA*-derived variations: 120 bp deletion [c.11435_11524+30del, p.(Gly3812Phefs*11)] and missense variant [c.12174C>G, (p.Cys4058Trp)];

[d] Patient 14 was compound heterozygous for a gene conversion in one allele and a *TNXB*/*TNXA* fusion gene in other allele, characterized both by the "*TNXA*-derived" missense variant [c.12174C>G, p.(Cys4058Trp)];

[e] Patient 15 was compound heterozygous for a *TNXB*/*TNXA* fusion gene in one allele characterized by the 120 bp deletion [c.11435_11524+30del, p(.Gly3812Phefs*11)], and a missense variant in the other allele;

[f] Patient 18 was compound heterozygous for a nonsense variant and a *TNXB*/*TNXA* fusion gene characterized by the *TNXA*-derived variations: 120 bp deletion [c.11435_11524+30del, p.(Gly3812Phefs*11)], and missense variant [c.12174C>G, (p.Cys4058Trp)];

[g] Patients 19 and 20 were compound heterozygous for a nonsense variant in one allele and a gene conversion in the other allele, characterized by the 120 bp deletion [c.11435_11524+30del, p.(Gly3812Phefs*11)].
